# Whole-body cryostimulation exposures effectively alleviates menstrual-related pain and associated sleep disturbances in young women: a randomized controlled trial

**DOI:** 10.3389/fpain.2025.1614153

**Published:** 2025-07-24

**Authors:** Quentin Bretonneau, Coralie Arc-Chagnaud, Benoit Dugué, Olivier Dupuy, Nathalie Delpech, Carina Enea, Laurent Bosquet

**Affiliations:** Laboratory MOVE – UR20296 – University of Poitiers, Poitiers, France

**Keywords:** menstrual cycle, pain, sleep quality, whole-body cryostimulation, well-being

## Abstract

**Introduction:**

Menstrual-related pain and sleep disturbances are widespread in women experiencing premenstrual syndrome and primary dysmenorrhea. Such disturbances could be alleviated through repeated whole-body cryostimulation (WBC) sessions. Therefore, this study aimed to assess the effect of menstrual-related pain on sleep parameters, and the impact of WBC exposures on pain and sleep.

**Materials and methods:**

Pain and sleep were evaluated for two 5-day periods under different conditions (control vs. WBC), randomly assigned across two consecutive menstrual cycles. Measurements began when the first pain/symptom indicating the onset of the menstrual phase was experienced. Pain was rated using a scale, while sleep was assessed using accelerometers and questionnaires. Throughout the 5-day WBC exposure, women underwent 3-min exposure to intense ventilated cold air each evening. After data collection, participants were categorized into high (HP) or low/no pain (LP) groups based on control pain scores.

**Results:**

Twenty-nine naturally menstruating women were assessed. Perceived sleep quality was lower in the HP group compared to the LP group during the control condition (Spiegel score: 20.1 ± 2.3 vs. 22.3 ± 1.9, respectively; Cohen's *d* = 1.1). Across both groups, perceived sleep quality improved with the number of WBC exposures (night1: 19.5 ± 3.2 vs. night5: 23.5 ± 3.8; Hedge's *g* = 1.10). In the HP group, pain was reduced in the WBC condition compared to the control condition. Changes in pain and perceived sleep quality following WBC were correlated (*r* = −0.86).

**Discussion:**

Women experiencing higher menstrual-related pain reported poorer perceived sleep quality. Their pain was reduced by WBC exposures. This improvement was highly associated with the enhancement in sleep quality.

## 1 Introduction

Premenstrual syndrome (PMS) and primary dysmenorrhea (PD) are two common menstrual cycle-related complaints among women of reproductive age (1–6). Specifically, PMS is characterized by emotional, behavioural, and physical symptoms that predominantly occur during the late-luteal phase and subside with the onset of menstruation. PD is described as painful uterine cramps not associated with organic diseases, typically starting just before or concurrent with menstrual flow (4, 6, 7). In women suffering from these gynaecological disorders, pain seems to be one of the most commonly experienced symptoms, particularly in the lower back and/or abdominal regions (1, 5).

In addition to pain, women suffering from PMS and PD also experience sleep disruption (7). In the study by Baker et al. (8), women with dysmenorrhea exhibited significant differences compared to those without menstrual pain in terms of sleep architecture, nocturnal body temperature, and circulating hormones. However, the impacts of PMS and PD on sleep are still not fully understood. In women with PMS, several studies have highlighted an alteration in perceived sleep quality without observing any concomitant objective deterioration through polysomnography (9–12).

Furthermore, previous studies have reported elevated levels of inflammation in women with PMS and PD (13–15). Additionally, inflammation has been shown to be associated both with pain (15) and sleep disturbances (16, 17). Therefore, in women experiencing PMS and PD, the pain and sleep disturbances could potentially be influenced by inflammation.

The most prevalent approach to alleviate discomfort associated with the menstrual cycle is medication, often used without prior consultation with healthcare professionals (1, 18). Additionally, strategies such as the use of contraceptive pills or dietary supplements, as well as the application of heat to the painful area, are also commonly employed (4, 6, 18, 19). Two recent studies have indicated that applying cold to the painful area may also be a promising approach for reducing this type of pain (20, 21).

Body cryostimulation is a technique involving exposure of the entire or nearly entire subject's body to very cold air (−60 °C to −180 °C) for a brief period (3–5 min) (22). It is recognized for its manifold positive effects on parasympathetic activity (23), tissue oxygenation (24), feelings of well-being, and sport performances (25). Additionally, body cryostimulation is renowned for its beneficial effects on inflammatory processes and pain (26). Recent studies have also demonstrated its beneficial effects on sleep (27–29), as well as on the cardiovascular, immune and nervous system (22, 30).

Therefore, body cryostimulation could be particularly beneficial for women experiencing menstrual pain and sleep disturbances before and during their periods. Hence, this study aimed to assess (1) the effect of menstrual-related pain on objective and self-reported sleep parameters, (2) the impact of repeated whole-body cryostimulation (WBC) exposures on pain and sleep, and the association between WBC-induced changes in pain and sleep. We hypothesized that women experiencing menstrual pain before and/or during their periods would exhibit lower sleep quantity and quality compared to those experiencing minimal or no pain. Additionally, it was postulated that WBC would lead to pain reduction and improved sleep, especially in women experiencing pain, and that changes in pain and sleep induced by WBC would be closely related.

## 2 Materials and methods

### 2.1 Experimental design

**Figure 1:**
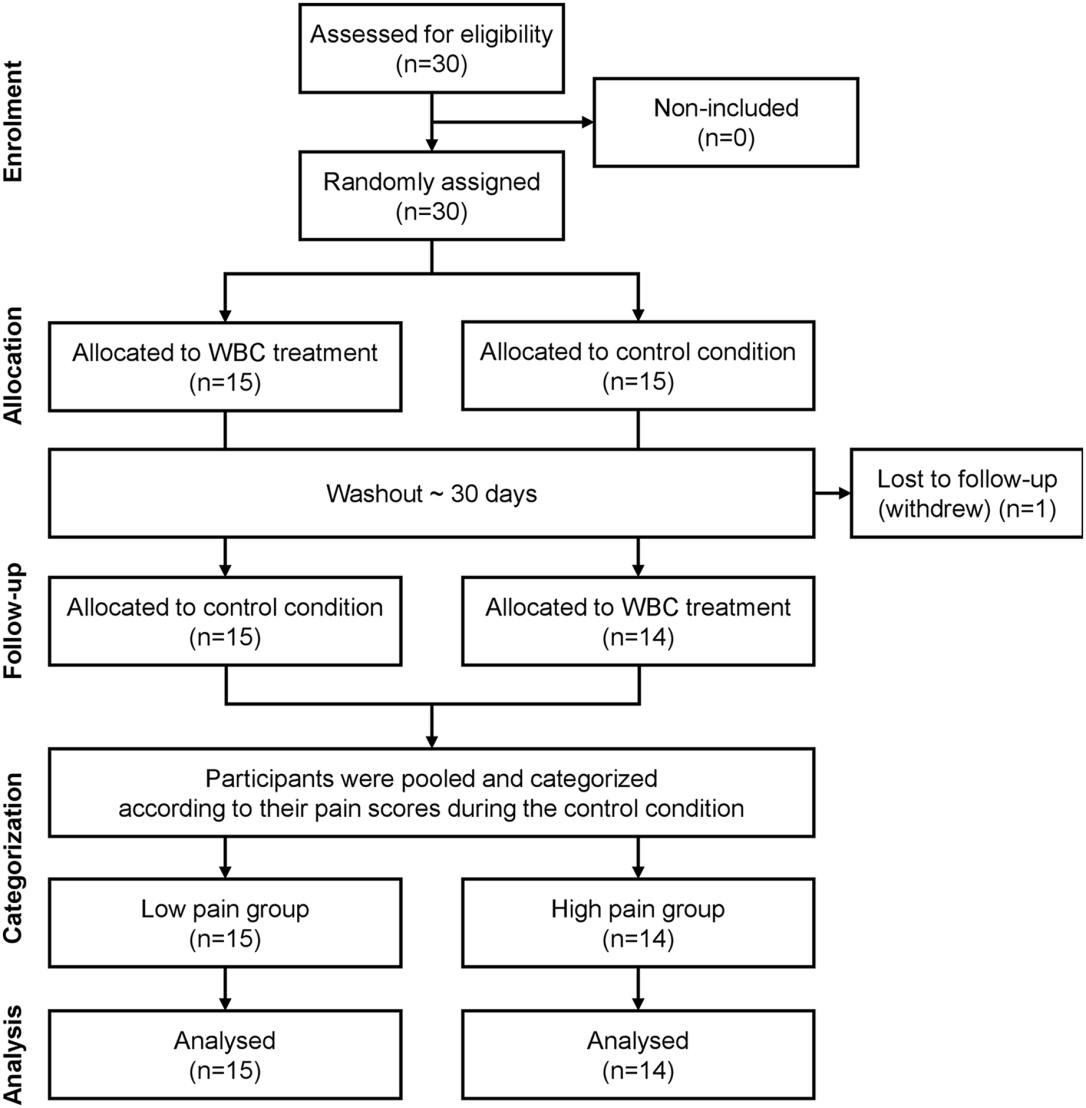
CONSORT flow diagram of the study (randomized controlled trial of non-pharmacological treatment).

This study employed a counterbalanced crossover randomized controlled design. Each participant underwent two 5-day assessment periods under two different conditions (control vs. WBC), with the order of conditions randomly assigned across two consecutive menstrual cycles (Figure 1).

### 2.2 Study population

#### 2.2.1 Sample size

Assuming an effect size of 0.5, considered reasonable based on the findings of Silva et al. (21), an *a priori* power analysis indicated that a sample size of 27 participants would be required to detect a beneficial effect of WBC on menstrual-related pain (*α* = 0.05; power = 0.80). To account for potential dropouts, the recruitment target was set at 30 participants (+10%).

#### 2.2.2 Recruitment

Participants were recruited through volunteer sampling following a public advertisement of the study, which outlined the main inclusion and non-inclusion criteria. Individuals who expressed interest were invited to attend an initial visit at the MOVE laboratory of the University of Poitiers, during which all inclusion and non-inclusion criteria were assessed by the experimenters.

#### 2.2.3 Inclusion and non-inclusion criteria

The inclusion criteria for this study were naturally menstruating women aged 18–35 years with regular menstrual cycles, defined as cycles lasting between 21 and 35 days over the past three months (31). Participants were required to have no legal guardianship or curatorship and no subordination. They had to be beneficiaries of a Social Security system, either personally or through a third party. Informed consent was obtained from each participant after a thorough and transparent explanation of the study.

The non-inclusion criteria included irregular menstrual cycles, any diagnosed gynaecological disorders (e.g., endometriosis, polycystic ovary syndrome), current use of analgesic or anti-inflammatory medications, pregnancy or breastfeeding, and specific contraindications for cryostimulation (30). Contraindications included hypertension, arteritis, recent myocardial infarction (within the past six months), stroke, and pulmonary embolism. Participants with respiratory conditions such as asthma or chronic bronchitis, circulatory insufficiency (e.g., Raynaud's syndrome), angina pectoris, or those with pacemakers or subcutaneous medical devices were also non-included in the study.

#### 2.2.4 Randomization

Randomization was conducted by the experimenters using an online tool that generated a sequence of fifteen “1” and fifteen “2”, assigned randomly (e.g., 2, 1, 1, 2, 1, 2…). Based on the order of inclusion, each participant was assigned the corresponding value in the sequence (i.e., the *n*th participant received the *n*th value), which determined the condition order for their participation: a value of “1” indicated that the participant would complete the control condition first, followed by the experimental condition. In contrast, a value of “2” indicated the reverse order.

#### 2.2.5 Pain categorization

To determine whether the effect of WBC on menstrual-related pain was influenced by the initial level of pain intensity, participants were classified *post hoc* into two groups according to their level of pain reported during the control condition: a low/no pain (LP) group and a high pain (HP) group (Figure 1).

### 2.3 Measurements

**Figure 2:**
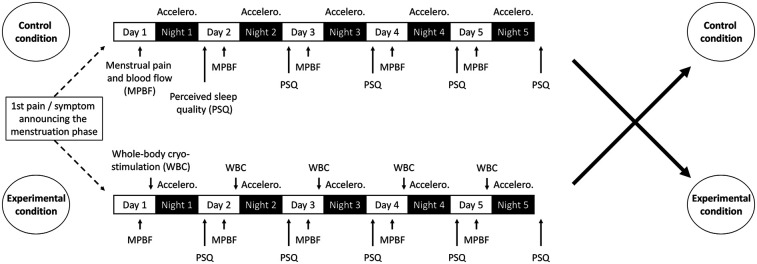
Experimental design. With Accelero., accelerometer.

Menstrual pain, menstrual flow and sleep were assessed upon the first report of pain or symptoms signalling the onset of the menstrual phase (Figure 2).

#### 2.3.1 Menstrual pain and flow

The overall intensity of pain experienced over the day (i.e., the primary outcome) was assessed using a 0–5 verbal rating scale (VRS), where 0 signified “No pain” and 5 indicated “Unbearable pain” (32). This variable was used both as a dependent variable (for monitoring daily pain) and as an independent variable for categorizing participants (LP group: mean VRS score <2.5; HP group: mean VRS score >2.5.).

Additionally, menstrual blood flow was assessed daily using the Pictorial Blood Loss Assessment Chart developed by Higham et al. (33). This questionnaire queries the amount of blood on sanitary pads and/or tampons, the number of hygienic protections used throughout the day, and the presence of clots along with their size.

#### 2.3.2 Sleep quantity and quality

##### 2.3.2.1 Self-reported sleep parameters

Perceived sleep quality was assessed every morning using the 6-item Spiegel questionnaire [0–30 score range; Spiegel (34)]. A score below 18 indicated sleep disturbances, and a score below 15 suggested severe alertness. A few minutes after getting up, the participants had to complete the questionnaire, which was integrated into their sleep diary. They also had to specify the bedtime (i.e., when they went to bed, not necessarily to sleep), the lights-out time (i.e., when they turned off lights or their smartphone intending to sleep) and the wake-up time

##### 2.3.2.2 Objective sleep parameters

Every night during the analysis periods, participants were required to wear an accelerometer on the non-dominant wrist to quantify their movements (ActiGraph wGT3X-BT, ActiGraph LLC, Pensacola, USA). Counts were recorded on the three-dimensional axis. Parameters including sleep efficiency, latency, number and duration of awakenings, total sleep time and sleep fragmentation index were also assessed (35).

### 2.4 Whole-body cryostimulation

In the WBC condition, the first cold exposure took place on the same evening as the initial report of pain or symptoms indicating the onset of the menstrual phase. Subsequently, participants underwent cold exposure every evening for the following four consecutive days (Figure 2). Each WBC session consisted of a 30-s exposure in a first ventilated cold chamber at −25 °C (perceived temperature: −50 °C) and a 3-min exposure in a second ventilated cold chamber at −70 °C (perceived temperature: −110 °C) (Aurore Concept, Noisiel, France). Participants entered the cryo-chamber wearing gloves, slippers, underwear, a cap and a surgical mask. Throughout the exposure period, participants were closely supervised by the researcher team. They were asked about the occurrence of any adverse effects (e.g., headache, dizziness, burning sensations, numbness, erythema, skin irritation, frostbite, or persistent and/or recurrent shivering during the evening following the exposure) immediately after each session and again the following day, prior to the next exposure. Additionally, they were instructed to avoid physical activity after the WBC session to avoid disrupting the effect of cold exposure.

### 2.5 Ethical considerations

The protocol adhered to the Helsinki Declaration and received approval from an ethics committee (IRB00012476-2023-31-01-223). All volunteers provided written consent before beginning the protocol.

### 2.6 Statistics

Standard statistical methods were used to calculate means and standard deviations. Gaussian distribution was verified using the Shapiro–Wilk test. A Student's *t*-test was performed to test the null hypothesis that sleep parameters and menstrual blood flow were not different between groups (low pain group vs. high pain group) in control condition. A two-way factorial analysis of variance (group × condition) with repeated measures on the condition factor (control vs. WBC) was performed to test the null hypothesis that measures were not different between groups and conditions. A three-way factorial analysis of variance (group × condition × time) with repeated measures on both condition and time factors (Day 1 to Day 5, or Night 1 to Night 5) was performed to test the null hypothesis that measures were not different between groups, conditions and times. Compound symmetry, or sphericity, was assessed using the Mauchly's test. When sphericity assumption was violated, F-ratios were adjusted using the Greenhouse–Geisser procedure if the epsilon correction factor was <0.75, or the Huynh–Feldt procedure if the epsilon correction factor was >0.75, to control for type I errors. Multiple comparisons were performed using the Tukey *post hoc* test. Effect sizes were evaluated using Hedges' *g* (*g*) and Cohen's *d* (*d*), categorized as small (0.2 < *d* or *g* < 0.5), moderate (0.5 < *d* or *g* < 0.8), or large (*d* or *g* > 0.8). Pearson's product-moment correlation was used to test the null hypothesis of an absence of association between variables or their variations. The Cook's distance was used once to identify and remove an outlier. Correlations over 0.90 were considered very high, between 0.70 and 0.89 were considered high, and between 0.50 and 0.69 were considered moderate (36). For data retention, participants who attended fewer than 80% of the WBC sessions were non-included in the data analysis. The significance level for all analyses was set at *p* < .05. All the calculations were conducted using Statistica (StatSoft, Tulsa, USA) and Excel (Microsoft, Redmond, USA).

## 3 Results

### 3.1 Participants

Thirty women with regular menstrual cycles were enrolled in the study. One participant withdrew, resulting in a final sample of twenty-nine women who completed the entire protocol (age: 22 ± 4 years; height: 1.60 ± 0.10 m; body mass: 60.3 ± 9.9 kg; body mass index: 26.7 ± 7.2 kg·m^−2^; body fat percentage: 22.5 ± 3.6%). The first participant was recruited in January 2023, and the last participant was recruited in March 2023. Data were collected from January 2023 to April 2023. Fourteen participants completed the protocol starting with the control condition, followed by the experimental condition, while fifteen participants completed the protocol in the reverse order. Among the participants, 48.3% were not using any form of contraception, 37.9% used oral contraceptives, 10.3% used a copper intrauterine device, and 3.5% used a contraceptive ring.

At the end of the study, the sample was divided into two groups based on the pain level measured during the control condition, resulting in a low pain (LP) group comprising 15 participants and a high pain (HP) group comprising 14 participants. The characteristics of the HP and LP groups are presented in Table 1. Anthropometric parameters did not differ significantly between the groups.

**Table 1:** Characteristics of low pain and high pain groups.

Characteristics	Low pain group *n* *=* *15*	High pain group *n* *=* *14*
Anthropometry		
Age (years old)	22.9 ± 3.5	23.5 ± 3.8
Body height (m)	1.66 ± 0.05	1.62 ± 0.06
Body mass (kg)	62.2 ± 10.0	58.2 ± 9.8
Body mass index (kg m^−2^)	26.7 ± 7.7	26.6 ± 6.8
Body fat (%)	22.6 ± 3.6	22.4 ± 3.7
Contraception		
No contraception (%)	47	50
Hormonal contraception		
*Oral contraceptive (%)*	53	21
*Contraceptive ring (%)*	—	7
Non-hormonal contraception		
*Copper intrauterine device (%)*	—	21

Anthropometric characteristics did not differ significantly between groups, *p* > 0.05.

### 3.2 Context

During the control and WBC conditions, menstruation appeared 2.5 ± 1.1 and 2.4 ± 1.2 days after the first pain/symptom announcing the onset of the menstrual phase, respectively (*p* > 0.05). In both conditions, approximately 25% of participants experienced menstruation on the first day of pain/symptom, 40% two days after, 80% three days after and 100% four and five days after. In 75% of cases, the time elapsed between the onset of pain/symptoms and the onset of menstruation was identical in both conditions. This percentage increased to 93% when a difference of ±1 day was tolerated. Cold exposures were conducted at 19:41 ± 1:17 with no significant difference between the 5 exposures. Additionally, cold exposures were conducted at 19:50 ± 1:25 for participants assigned to the WBC condition before the control condition, and at 19:32 ± 1:09 for those assigned to the WBC condition after the control condition (*p* > 0.05).

### 3.3 Pain

**Figure 3:**
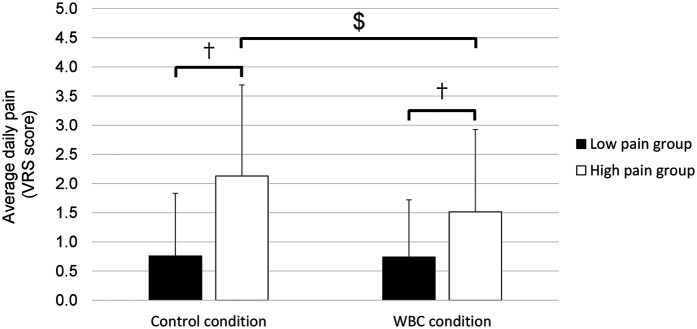
Average daily pain in low pain and high pain groups in control and whole-body cryostimulation (WBC) conditions. †, difference between groups (*p* < 0.01). $, difference between conditions (*p* < 0.01).

As shown in Figure 3, pain experienced during both the control and WBC conditions was higher in the HP group compared to the LP group (*p* < 0.05; Cohen's *d* = 2.3). Furthermore, in the HP group, pain was lower in the WBC condition than in the control condition (*p* < 0.05; Hedges' *g* = 0.8).

### 3.4 Menstrual flow

During the control condition, the Higham score was higher in the HP group than in the LP group (30 ± 25 vs. 10 ± 7, *p* < 0.05; Cohen's *d* = 1.1). Across both groups, this score tended to decrease with WBC exposures (from 20 ± 29 in the control condition to 15 ± 27 in the WBC condition, *p* = 0.07).

### 3.5 Sleep

**Figure 4:**
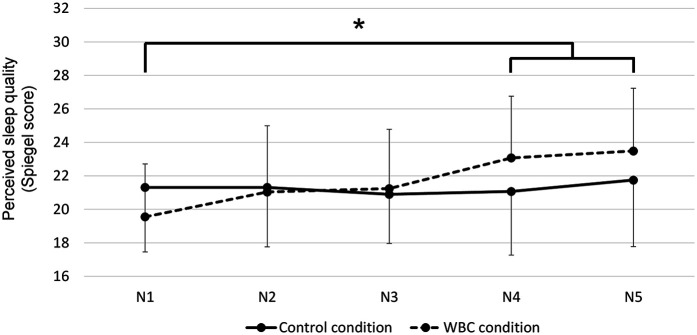
Night-to-night perceived sleep quality in control and whole-body cryostimulation (WBC) conditions. *p < 0.001 vs. the first night for the WBC condition.

No significant differences were observed between groups, conditions and times for sleep parameters assessed by accelerometry. In contrast, the Spiegel score was significantly lower in the HP group than in the LP group in control condition (20.1 ± 2.3 vs. 22.3 ± 1.9 respectively; Cohen's *d* = 1.1). Across both groups, the Spiegel score was higher during the last two nights of the WBC condition than during the first night (*p* < 0.05; Hedge's *g* = 1.10; Figure 4).

### 3.6 Pain and sleep relationship

**Figure 5:**
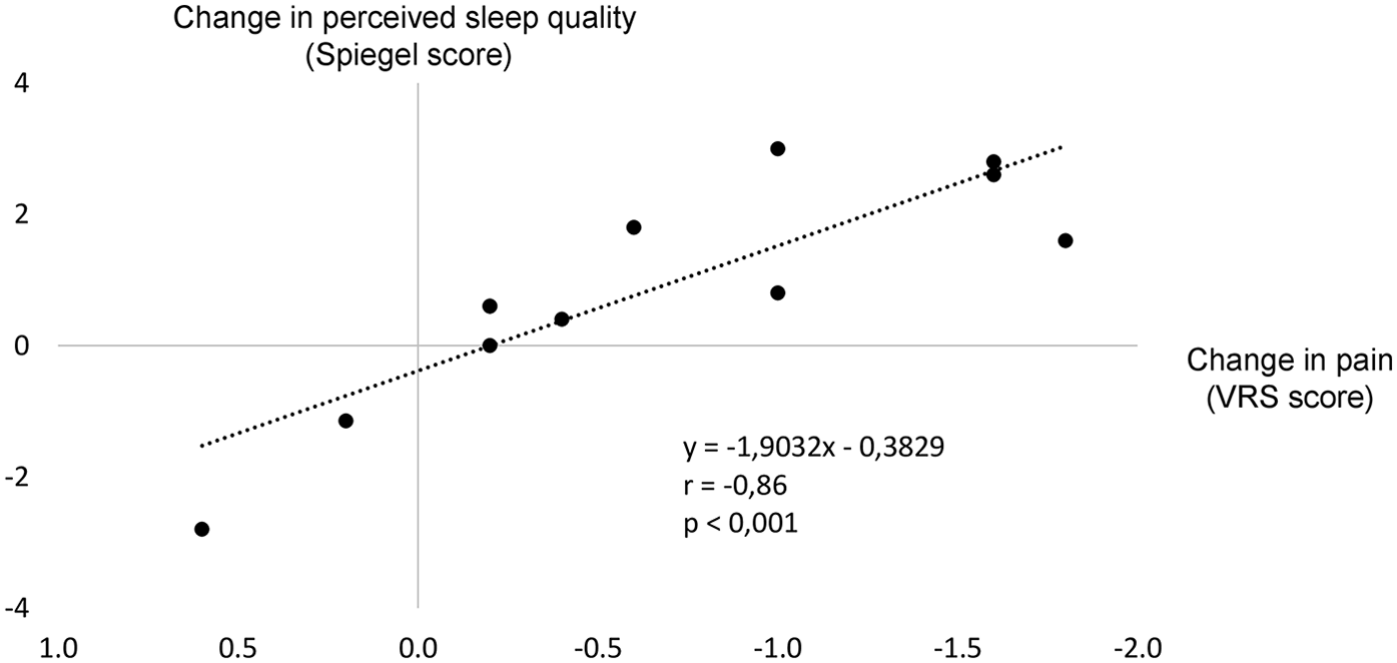
Level of association between the changes (Δ) in pain and perceived sleep quality from the control condition to the whole-body cryostimulation (WBC) condition (Δ, value in the WBC condition—value in the control condition).

In the HP group, changes in pain and perceived sleep quality from the control condition to the WBC condition were associated (Figure 5).

## 4 Discussion

The primary purpose of this study was to assess the effect of menstrual-related pain on sleep. In this context, objective and self-reported sleep parameters were assessed in two groups of women experiencing high and low/no pain. The results indicated that women with higher levels of pain reported poorer perceived sleep quality. The second purpose of this study was to assess the effect of repeated WBC exposures on pain and sleep. As the WBC exposures progressed, there was a corresponding improvement in perceived sleep quality. Furthermore, we observed that menstrual-related pain was reduced during the WBC exposures among women who typically experience high levels of pain. For this group, we also observed an association between the reduction in pain and the improvement in perceived sleep quality induced by WBC.

### 4.1 Menstrual-related pain and sleep

As shown by Baker et al. (8), sleep may be particularly disturbed in women experiencing severe PMS- or PD-related pain. These authors found that women with painful menstruation exhibited poorer perceived sleep quality, lower sleep efficiency, and shorter rapid-eye movement (REM) sleep compared to women without PD. Additionally, during the menstrual phase, where the pain was at its highest level, women with PD reported poorer perceived sleep quality and lower sleep efficiency compared to their own no-painful luteal and follicular phases, thereby underlying the potential role of menstrual-related pain in sleep disturbances. In this population, Çaltekin et al. (11) recently reported a correlation between pain and sleep disorders. In the current study, we observed that women who typically experience pain also exhibit poorer sleep quality than their counterparts who experience low/no pain, aligning with previous findings. However, this effect of pain on sleep was observed in subjective parameters, but not in those objectively assessed using accelerometery. This discrepancy has already been reported in previous studies (7, 9–12, 37), and can be explained by the fact that subjective and objective measures may assess different aspects of sleep (38).

The relationship between menstrual-related pain and sleep may be mediated by inflammation, which can be particularly elevated in women with PMS and PD (13, 18). In their study, Gold et al. (15) demonstrated the inflammation-pain relationship by showing that an elevated C-reactive protein (CRP) blood concentration (>3 mg/L) was significantly associated with a 40% increased odds of reporting menstrual-related abdominal cramps/back pain. On their side, Ditmer et al. (39) emphasized the inflammation-sleep relationship by demonstrating that inflammation—especially high levels of interleukin-1 beta and tumor necrosis factor alpha—may disrupt sleep architecture by promoting non-REM sleep at the expense of REM sleep. These findings are consistent with those presented in the meta-analysis by Irwin et al. (16), which demonstrated a strong association between levels of inflammation (CRP and interleukin-6) and sleep disturbances.

### 4.2 Effect of whole-body cryostimulation exposures on menstrual-related pain, sleep and their relationship

The secondary purpose of this study was to assess the effect of WBC exposures on menstrual-related pain, sleep and their relationship. According to our results, this innovative non-pharmacological approach, involving exposure of the body to stimulating cold thermal stress, effectively reduces pain in healthy young women who typically experience it before or during menstruation. This finding aligns with those observed in recent studies by de Araújo et al. (20) and Silva et al. (21), where menstrual-related pain was alleviated by applying an ice-pack during three consecutive days on the painful area (back or abdominal region). In addition, we observed a nearly significant decrease in the Higham score during the WBC period compared to the control period, suggesting a beneficial effect of intense cold exposure on menstrual flow and its management. This benefit could be explained by a reduction in uterine contractions responsible for a significant flow at the beginning of menstruation. This hypothesis is supported by the decrease in prostaglandin E2 concentration reported by Banfi et al. (40) in response to cryostimulation.

Furthermore, our study demonstrated that a daily WBC exposure near bedtime for five days effectively enhances perceived sleep quality before or during menstrual periods, irrespective of pain severity during this time. In previous studies conducted in active subjects or in athletes, subjective sleep parameters improved after one exposure (27, 28), or after daily exposure during several days (41). Thus, our results are consistent with the scientific literature. However, Douzi et al. (27, 28) also showed a benefit of body cryostimulation on objective sleep parameters assessed by accelerometry, a result we did not observed in the current study. It must be noted that only males were investigated in these previously cited works, and male and female participants may react differently to WBC.

Additionally, we observed a strong correlation between the reduction in pain and the improvement in sleep quality induced by WBC. In a previous study by Iacovides et al. (42), improvements in subjective and objective sleep parameters were observed after a reduction in pain induced by the administration of non-steroidal anti-inflammatory drugs in women with PD, but the authors did not described the relationship between these changes.

### 4.3 Mechanisms underlying the benefits of cryostimulation on pain, sleep and their relationship

The reduction in menstrual-related pain observed during WBC exposures may be attributed to a deceleration of pain signals to the brain. Indeed, a reduction in the conduction velocity of tibial nerve fibers was observed by Algafly and George (43) following cold application to the ankle. These authors also noted an associated increase in pain threshold and pain tolerance, which may provide an additional explanation for the benefits of WBC observed in our study. Additionally, cryostimulation may reduce pain perception through the production of analgesic beta-endorphin and noradrenaline (44, 45).

Moreover, the benefits of WBC on sleep could imply thermoregulatory processes. Indeed, following cryostimulation exposure, core body temperature decreases (46, 47). In this context, the natural night-time decline in core body temperature could be accelerated and/or intensified after WBC, especially when the exposure is scheduled in the evening as in the current study. This could then improve sleep onset and quality (48, 49). However, additional studies are needed to clarify the links between WBC-induced changes in core body temperature and sleep quality.

Lastly, the primary mechanism that can explain the association between pain reduction and improved sleep quality induced by WBC likely involves the inflammatory processes. Indeed, as previously described, pain and sleep could be mediated by inflammation and repeated body cryostimulation exposures have been shown to effectively reduce the activity of pro-inflammatory cytokines and to increase the activity of anti-inflammatory cytokines (50). Therefore, analyzing blood inflammatory markers would be an interesting perspective to validate the hypothesis that WBC-induced improvements in pain and sleep are mediated by changes in inflammatory level.

### 4.4 Methodological aspects of the study

Between the control and experimental conditions, we observed an interesting intra-individual consistency regarding the delay between the onset of menstrual-related symptoms and the onset of menstruation. This result suggests that the difference we observed in perceived sleep quality along with the WBC exposures was probably not related to earlier or later menses from the onset of symptoms. This hypothesis is reinforced by the fact that Baker and Driver (51) did not find significant difference in perceived sleep quality between the premenstrual and menstrual phases in healthy young women.

Furthermore, precautions were taken to ensure consistent daily exposure to cryostimulation and between randomized trials (women exposed to cryostimulation before vs. after the control condition). Moreover, we did not observe significant difference regarding the timing of cryostimulation exposure. Thus, the difference we observed in perceived sleep quality along with the WBC exposures was probably not related to a variation in the timing of WBC session throughout the treatment period.

### 4.5 Limits of the study and possible improvements

One of the main limitations of this study is that it examined the effects of WBC on menstrual-related pain and sleep only during the WBC treatment period, and not in the post-treatment phase. As a result, it is difficult to determine, based on the current data, whether these benefits are maintained over time. Future studies should consider evaluating the residual effects of WBC on these parameters.

### 4.6 Conclusion and perspectives

Whole-body cryostimulation appears particularly effective in reducing menstrual-related pain and improving sleep quality. For women experiencing these issues, this innovative and non-pharmacological approach could significantly enhance well-being and quality of life before, during, and after menses. Consequently, such an approach could present socio-educational and socio-economic benefits by reducing, for instance, absenteeism in schools and workplaces, a genuine consequence of menstrual pain (52). Among athletes, where 50% of women suffer from PMS (2), cryostimulation exposures could also reduce training absenteeism, which is critical for achieving high-level performance.

## Data Availability

The raw data supporting the conclusions of this article will be made available by the authors upon request, within a clearly defined framework provided by the requester, as long as they are not used for personal, professional, or commercial purposes.
